# Finding *Aedes aegypti* in a natural breeding site in an urban zone, Sao Paulo, Southeastern Brazil

**DOI:** 10.1590/S1518-8787.2016050006245

**Published:** 2016-03-01

**Authors:** Tamara Nunes Lima-Camara, Paulo Roberto Urbinatti, Francisco Chiaravalloti-Neto

**Affiliations:** Departamento de Epidemiologia. Faculdade de Saúde Pública. Universidade de São Paulo. São Paulo, SP, Brasil

**Keywords:** *Aedes*, growth & development, Disease Vectors, Vector Control

## Abstract

This is the description of how nine *Aedes aegypti* larvae were found in a natural breeding site in the Pinheiros neighborhood, city of Sao Paulo, SP, Southeastern Brazil. The record was conducted in December 2014, during an entomological surveillance program of dengue virus vectors, with an active search of potential breeding sites, either artificial or natural. Finding *Ae. aegypti* larvae in a tree hole shows this species’ ability to use both artificial and natural environments as breeding sites and habitats, which points towards the importance of maintaining continuous surveillance on this mosquito in all kinds of water-holding containers.

## INTRODUCTION

*Aedes (Stegomyia) aegypti* (Linnaeus, 1762) is considered a mosquito of great importance to public health care since the early 20th century, when it was found to be a vector of urban yellow fever. Currently, *Ae. aegypti* is considered the main vector of the four dengue virus serotypes circulating in Brazil (DENV-1, DENV-2 DENV-3, and DENV-4), besides being a competent enough vector to transmit the arboviruses chikungunya and Zika, which have just arrived in the country^5^.

The behavior of this vector is highly endophilic and anthropophilic; it seeks shelter inside houses, where it feeds from human blood. Thus, *Ae. aegypti* is most frequently found in urban and sub-urban environments, with high concentrations of human beings and houses. Present in all Brazilian states, the females of this species often lay their eggs in rainwater deposits such as artificial containers or objects for domestic use, such as tires, cans, bottles, flower pots, water reservoirs, barrels, vats, abandoned swimming pools and aquariums, among others1. However, albeit rarely, some studies report the finding of *Ae. aegypti* larvae in bromeliad plants in urban areas^2^ ^,^ ^4^.

No efficient vaccines are currently available against dengue fever, chikungunya, or Zika. Thus, the only controllable element in the epidemiological chain of these arboviruses is the mosquito, and the main strategy to control that vector mainly includes eliminating its potential breeding sites^3^.

This study aimed to report the finding of *Ae. aegypti* larvae in a natural breeding site in an urban area of the city of Sao Paulo, during entomological surveillance procedures.

## METHODS

An active search for *Ae. aegypti* immatures was conducted in both artificial and natural breeding sites on December 3, 2014, in an urban area located in the Pinheiros neighborhood, city of Sao Paulo, SP, Southeastern Brazil, which is a very busy area.

Plastic ladles with maximum capacity of 80 ml were used to collect the immatures. The material collected with the ladles was transferred to plastic containers labeled according to the types of breeding sites, and then sent to an entomology laboratory. The collected larvae were identified under a stereo microscope, according to the dichotomous key proposed by Consoli and Lourenço de Oliveira^1^ (1994).

## RESULTS

Nine larvae in the L4 stage were collected in a hole of a tree that belonged to the Moraceae family and to the Ficus genus (Figure, A and B). All nine larvae were identified as *Ae. aegypti* and kept in a laboratory until they fully matured, when their species was confirmed.

**Figure:**
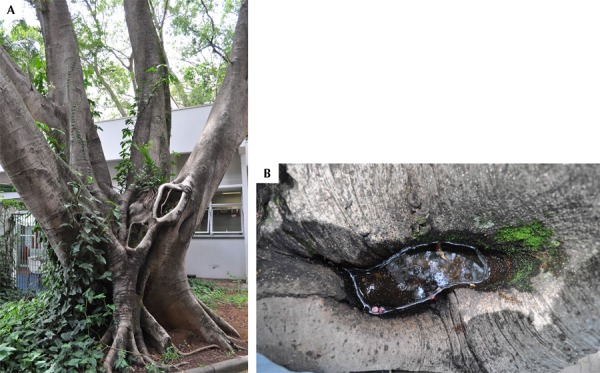
Natural breeding site with *Ae. aegypti* larvae. (A) Partial view of the tree located in a garden in the Pinheiros neighborhood, where the nine *Ae. aegypti* larvae were found. (B) Detail of the tree hole from where the larvae were collected.

## DISCUSSION

In Brazil, field studies with *Ae. aegypti* show that this mosquito is more frequently captured inside houses, and female mosquitoes are found to be engorged with human blood. That reinforces the endophilic and anthropophilic behaviors of that species^1^. Besides that, finding *Ae. aegypti* in immature stages is more frequent in artificial breeding sites in urban and sub-urban areas, although larvae of this species have already been found in natural breeding sites, such as bromeliad plants^2^ ^,^ ^4^.

This study reports the finding of nine *Ae. aegypti* larvae in a tree hole near some houses, during a search for potential breeding sites of this vector in the area. Finding L4 stage *Ae. aegypti* larvae in a natural breeding site points towards two important aspects. The first is that entomological surveillance on this species in artificial containers may have been efficient, as the reduced availability of that type of breeding site causes *Ae. aegypti* to seek other places to lay its eggs. The second aspect is the confirmed plasticity of this vector, which, in the absence of artificial breeding sites, lays its eggs in natural breeding sites, a behavior that is not very usual for this species.

In conclusion, it is necessary to maintain entomological surveillance on *Ae. aegypti* not only in artificial containers but also in natural water-holding surfaces, even in urban areas, to keep outbreaks of arboviruses such as dengue, chikungunya, and Zika controlled.
