# Supplementary material for: Finding Aedes aegypti in a natural breeding site in an urban zone, Sao Paulo, Southeastern Brazil
Source: Rev Saude Publica. 2016 Mar 1;50:3. doi: 10.1590/S1518-8787.2016050006245 (PMC4794767; doi:10.1590/S1518-8787.2016050006245)
Supplement: Translation

# Translation: Encontro de *Aedes aegypti* em criadouro natural de área urbana, São Paulo, SP, Brasil

**Authors:** Tamara Nunes Lima-Camara, Paulo Roberto Urbinatti, Francisco Chiaravalloti-Neto

**Affiliations:** Departamento de Epidemiologia. Faculdade de Saúde Pública. Universidade de São Paulo. São Paulo, SP, Brasil

**Keywords:** *Aedes*, crescimento & desenvolvimento, Vetores de Doenças, Reservatórios de Doenças, Controle de Vetores

## Abstract

Descreve-se o encontro de nove larvas de Aedes aegypti em criadouro natural no bairro de Pinheiros, município de São Paulo, SP. O registro ocorreu no mês de dezembro de 2014, durante programa de vigilância entomológica dos vetores do vírus dengue, com busca ativa de potenciais criadouros, artificiais ou naturais. O encontro de larvas de Ae. aegypti em oco de árvore revela a capacidade dessa espécie de utilizar tanto criadouros artificiais como naturais para o seu desenvolvimento, o que aponta para a importância da manutenção de vigilância contínua sobre esse mosquito em todos os tipos de recipientes que podem acumular água.

## INTRODUÇÃO

*Aedes (Stegomyia) aegypti* (Linnaeus, 1762) é considerado mosquito de grande importância para a saúde pública desde o início do século XX, quando foi incriminado como vetor da febre amarela urbana. Atualmente, *Ae. aegypti* é considerado o principal vetor dos quatro sorotipos do vírus dengue circulantes no Brasil (DENV-1, DENV-2 DENV-3 e DENV-4), além de apresentar elevada competência vetorial para transmitir os arbovírus chikungunya e Zika, recém-emergidos no País^5^.

O comportamento desse vetor é altamente endofílico e antropofílico; ele utiliza o interior das casas para se abrigar e alimentar-se do sangue humano. Dessa forma, o encontro de *Ae. aegypti* ocorre, principalmente, em ambientes urbanos e suburbanos, onde há elevada concentração populacional humana e de residências. Presente em todos os estados brasileiros, as fêmeas dessa espécie frequentemente depositam seus ovos em recipientes artificiais com água da chuva ou de uso doméstico, como pneus, latas, vidros, garrafas, pratos de vasos, caixas d’água, tonéis, latões, piscinas e aquários abandonados, entre outros^1^. Entretanto, embora pouco comum, alguns estudos reportam o encontro de larvas de *Ae. aegypti* em bromélias localizadas em áreas urbanas^2^ ^,^ ^4^.

Atualmente, não estão disponibilizadas vacinas eficazes contra dengue, chikungunya e Zika. Assim, o único elemento da cadeia epidemiológica dessas arboviroses que pode ser controlado é o mosquito, e a principal estratégia de controle desse vetor envolve, principalmente, a eliminação dos seus criadouros potenciais^3^.

Este estudo teve como objetivo relatar o encontro de larvas de *Ae. aegypti* em criadouro natural de área urbana da cidade de São Paulo, durante procedimentos de vigilância entomológica.

## MÉTODOS

Em 3 de dezembro de 2014, foi realizada busca ativa de imaturos de *Ae. aegypti* em criadouros artificiais e naturais em área urbana localizada no bairro de Pinheiros, São Paulo, SP, com grande circulação de pessoas.

Para a coleta dos imaturos, foram utilizadas conchas plásticas, com capacidade máxima de 80 ml. O material coletado com as conchas foi transferido para vasilhas de plástico, etiquetadas de acordo com o tipo de criadouro, e enviadas para laboratório de entomologia. As larvas coletadas foram identificadas sob microscópio estereoscópico, seguindo chave dicotômica proposta por Consoli e Lourenço de Oliveira^1^ (1994).

## RESULTADOS

Nove larvas em estádio L4 foram coletadas em oco de árvore, pertencente à Família Moraceae e ao gênero *Ficus* (Figura, A e B). Todas as nove larvas foram identificadas como *Ae. aegypti* e mantidas em laboratório até a emergência dos adultos, quando foi feita a confirmação da espécie.

**Figura:** Criadouro natural com larvas de *Ae. aegypti*. (A) Vista parcial da árvore localizada em jardim no bairro de Pinheiros, onde foram encontradas as nove larvas de *Ae. aegypti*. (B) Detalhe do oco de árvore onde as larvas foram coletadas.

## DISCUSSÃO

No Brasil, os estudos realizados em campo com *Ae. aegypti* mostram que esse mosquito é mais frequentemente capturado no intradomicílio, com as fêmeas ingurgitadas apresentando sangue de origem humana no estômago. Isso reforça o comportamento endofílico e antropofílico da espécie^1^. Além disso, o encontro das formas imaturas de *Ae. aegypti* é mais frequente em criadouros artificiais de áreas urbanas e suburbanas, embora larvas dessa espécie já tenham sido registradas em criadouros naturais, como bromélias^2^ ^,^ ^4^.

Neste estudo, foi relatado o encontro de nove larvas de *Ae. aegypti* em oco de árvore em peridomicílio, durante busca por criadouros potenciais do vetor nessa área. O encontro de larvas de quarto estádio de *Ae. aegypti* em criadouro natural aponta para dois aspectos importantes. O primeiro deles é que a vigilância entomológica sobre essa espécie em recipientes artificiais pode estar sendo eficiente, pois a redução da oferta desse tipo de criadouro faz com que o *Ae. aegypti* busque outros locais para realizar a oviposição. O segundo aspecto é a comprovação da plasticidade desse vetor que, na ausência dos criadouros artificiais, deposita seus ovos em criadouros naturais, comportamento pouco frequente para a espécie.

Em conclusão, faz-se necessária a manutenção da vigilância entomológica do *Ae. aegypti* não só em recipientes artificiais, mas também em naturais, mesmo em áreas urbanas, a fim de serem controlados surtos de arboviroses, como dengue, chikungunya e Zika.
